# Bi-allelic variants in *SNF8* cause a disease spectrum ranging from severe developmental and epileptic encephalopathy to syndromic optic atrophy

**DOI:** 10.1016/j.ajhg.2024.02.005

**Published:** 2024-02-28

**Authors:** Melanie Brugger, Antonella Lauri, Yan Zhen, Laura L. Gramegna, Benedikt Zott, Nikolina Sekulić, Giulia Fasano, Robert Kopajtich, Viviana Cordeddu, Francesca Clementina Radio, Cecilia Mancini, Simone Pizzi, Graziamaria Paradisi, Ginevra Zanni, Gessica Vasco, Rosalba Carrozzo, Flavia Palombo, Caterina Tonon, Raffaele Lodi, Chiara La Morgia, Maria Arelin, Cristiane Blechschmidt, Tom Finck, Vigdis Sørensen, Kornelia Kreiser, Gertrud Strobl-Wildemann, Hagit Daum, Rachel Michaelson-Cohen, Lucia Ziccardi, Giuseppe Zampino, Holger Prokisch, Rami Abou Jamra, Claudio Fiorini, Thomas Arzberger, Juliane Winkelmann, Leonardo Caporali, Valerio Carelli, Harald Stenmark, Marco Tartaglia, Matias Wagner

**Affiliations:** 1 Institute of Human Genetics, Technical University of Munich, Munich, Germany; 2 Molecular Genetics and Functional Genomics, Ospedale Pediatrico Bambino Gesù, IRCCS, 00146 Rome, Italy; 3 Institute for Cancer Research, Oslo University Hospital, Oslo, Norway; 4 IRCCS Istituto Delle Scienze Neurologiche di Bologna, Programma Neuroimmagini Funzionali e Molecolari, Bologna, Italy; 5 Department of Biomedical and Neuromotor Sciences (DIBINEM), University of Bologna, Bologna, Italy; 6 Department of Diagnostic and Interventional Neuroradiology, Klinikum Rechts der Isar, Technical University of Munich, Munich, Germany; 7 Institute of Neuroscience, Technical University of Munich, Munich, Germany; 8 Centre for Molecular Medicine Norway (NCMM), Nordic EMBL Partnership, Faculty of Medicine, University of Oslo, Oslo, Norway; 9 Department of Chemistry, University of Oslo, P.O. Box 1033, Blindern, Norway; 10 Institute of Neurogenomics, Helmholtz Zentrum München, Neuherberg, Germany; 11 Dipartimento di Oncologia e Medicina Molecolare, Istituto Superiore di Sanità, Rome, Italy; 12 Unit of Muscular and Neurodegenerative Disorders and Unit of Developmental Neurology Piazza S. Onofrio 4, 00165 Rome, Italy; 13 Department of Neurorehabilitation and Robotics, Ospedale Pediatrico Bambino Gesù, IRCCS, Rome, Italy; 14 Translational Pediatrics and Clinical Genetics Research Division, Ospedale Pediatrico Bambino Gesù, IRCCS, Rome, Italy; 15 IRCCS Istituto Delle Scienze Neurologiche di Bologna, Programma di Neurogenetica, Bologna, Italy; 16 Department for Women and Child Health, Hospital for Children and Adolescents, University Hospitals, University of Leipzig, Leipzig, Germany; 17 Department of Neuropathology, Hospital Nürnberg, Nürnberg, Germany; 18 Department of Radiology and Neuroradiology, Rehabilitation and University Hospital Ulm, Ulm, Germany; 19 Praxis für Humangenetik, Ulm, Germany; 20 Department of Genetics, Hadassah Medical Organization and Faculty of Medicine, Hebrew University of Jerusalem, Jerusalem, Israel; 21 Department of Gynecology, Shaare Zedek Medical Center, Jerusalem, Israel; 22 Medical Genetics Unit, Shaare Zedek Medical Center, Jerusalem, Israel; 23 IRCCS-Fondazione Bietti, Rome, Italy; 24 Center for Rare Diseases and Birth Defects, Department of Woman and Child Health and Public Health, Fondazione Policlinico Universitario A. Gemelli IRCCS, Rome, Italy; 25 Università Cattolica Sacro Cuore, Rome, Italy; 26 Institute of Human Genetics, University Medical Center Leipzig, Leipzig, Germany; 27 Department of Psychiatry and Psychotherapy, University Hospital, Ludwig-Maximilians-University, Munich, Germany; 28 Center for Neuropathology and Prion Research, University Hospital Munich, Ludwig-Maximilians-University, Munich, Germany; 29 Munich Cluster for Systems Neurology (SyNergy), Munich, Germany; 30 Division of Pediatric Neurology, LMU Center for Development and Children with Medical Complexity, Ludwig-Maximilians-University Munich, Munich, Germany

**Keywords:** developmental and epileptic encephalopathy, leukoencephalopathy, ESCRT-II, autophagy, optic atrophy, neurogenetics

## Abstract

The endosomal sorting complex required for transport (ESCRT) machinery is essential for membrane remodeling and autophagy and it comprises three multi-subunit complexes (ESCRT I-III). We report nine individuals from six families presenting with a spectrum of neurodevelopmental/neurodegenerative features caused by bi-allelic variants in *SNF8* (GenBank: NM_007241.4), encoding the ESCRT-II subunit SNF8. The phenotypic spectrum included four individuals with severe developmental and epileptic encephalopathy, massive reduction of white matter, hypo-/aplasia of the corpus callosum, neurodevelopmental arrest, and early death. A second cohort shows a milder phenotype with intellectual disability, childhood-onset optic atrophy, or ataxia. All mildly affected individuals shared the same hypomorphic variant, c.304G>A (p.Val102Ile). In patient-derived fibroblasts, bi-allelic *SNF8* variants cause loss of ESCRT-II subunits. *Snf8* loss of function in zebrafish results in global developmental delay and altered embryo morphology, impaired optic nerve development, and reduced forebrain size. *In vivo* experiments corroborated the pathogenicity of the tested *SNF8* variants and their variable impact on embryo development, validating the observed clinical heterogeneity. Taken together, we conclude that loss of ESCRT-II due to bi-allelic *SNF8* variants is associated with a spectrum of neurodevelopmental/neurodegenerative phenotypes mediated likely via impairment of the autophagic flux.

## Teaser

We identified bi-allelic variants in *SNF8*, encoding a subunit of the ESCRT-II complex, in individuals presenting with a disease spectrum ranging from epileptic encephalopathy to syndromic optic atrophy—findings also present in a zebrafish model. Functional experiments suggest impaired autophagic flux as the disease mechanism.

## Introduction

Involution and scission of cellular membranes with negative curvature, as required during endosomal protein sorting and multivesicular body (MVB) formation, cytokinetic abscission, membrane repair, autophagosome closure, and many other cellular processes, are mediated by the conserved endosomal sorting required for transport (ESCRT) machinery.^1^ This machinery comprises three subcomplexes termed ESCRT I to III. ESCRT-III, which forms polymeric helical filaments, is thought to mediate the membrane scission activity of the ESCRT machinery in concert with the ATPase VPS4, whereas ESCRT-I and -II are required to recruit ESCRT-III to appropriate membranes. Given the importance of membrane dynamics in a wide range of cellular processes, it is not surprising that dysfunction of the ESCRT machinery gives rise to disease.^2^^,^^3^

The human ESCRT-II complex is a heterotetramer comprising one subunit each of SNF8 (EAP30/VPS22) and VPS36 (EAP45) and two subunits of VPS25, together forming a Y-like structure with SNF8 and VPS36 making the body of the Y in a side-by-side manner and VPS25 constituting the arms.^4^ ESCRT-II forms a stable complex in the cytosol and loss of any one subunit results in a reduction in the protein levels of the other subunits, indicating that only the fully assembled complex is stable.^5^^,^^6^ Within this complex, SNF8 does not participate in the binding of ESCRT-I or ESCRT-III complexes but interacts with the GLUE domain of VPS36 to facilitate membrane binding. The N-terminal portion of SNF8 possibly contains functional properties for cargo sorting.^7^

Knockdown of ESCRT-II in HeLa cells impairs degradation of cell surface proteins, such as the epidermal growth factor receptor (EGFR) and chemokine receptor CXCR4, via MVBs.^8^ Due to their retention in early endosomes, it has been suggested that loss of ESCRT-II blocks the trafficking of endocytosed cargo along the endosome/lysosome pathway likely by inhibiting the sorting of cargo to intraluminal vesicles in MVBs.^8^ However, there is evidence that the ESCRT-II complex is dispensable for degradation of some membrane proteins and thus ESCRT-II-dependent sorting for MVBs might be cargo specific.^6^

ESCRTs are also known to play a prominent role in autophagy—either by mediating the closure of the phagophore^9^^,^^10^^,^^11^ by facilitating in the fusion of autophagosomes with lysosomes,^12^^,^^13^ or by mediating trafficking of lysosomal enzymes to autolysosomes.^14^ However, the role of individual subunits has not yet been fully determined.

Knockout of several individual ESCRT subunits (ESCRT-I: *Tsg101*, *Vps37a*, *Vps37d*; ESCRT-II: *Vps25*, *Vps36*; ESCRT-III: *Chmp2b*, *Vps20*, *Chmp3*, *Chmp7*, *Ist1*, *Vps4a*) in mouse models results in early embryonic or preweaning lethality,^15^ indicating that loss of ESCRT functions is not compatible with healthy development. Consistent with this hypothesis, bi-allelic loss-of-function (LoF) variants affecting multiple ESCRT subunits, including all three subunits of ESCRT-II, are not present in the general population, as observed in the Genome Aggregation Database (gnomAD).^16^ To date, variants in a few of the genes encoding ESCRT subunits or associated proteins have been linked to Mendelian disorders, which are predominantly neurological diseases (*VPS37A*, spastic paraplegia 53, autosomal-recessive [MIM: 614898];^17^ *CHMP1A*, pontocerebellar hypoplasia, type 8 [MIM: 614961];^18^ *CHMP2B*, frontotemporal dementia and/or amyotrophic lateral sclerosis 7 [MIM: 600795];^19^ *PDCD6IP/ALIX*, microcephaly 29, primary, autosomal-recessive [MIM: 620047];^20^^,^^21^^,^^22^ *VPS4A*, CIMDAG syndrome [MIM: 619273]^23^).

We report nine individuals from six unrelated families presenting with bi-allelic *SNF8* (MIM: 610904) variants and a spectrum of neurodevelopmental/neurodegenerative phenotypes, including lethal developmental and epileptic encephalopathy with leukoencephalopathy, optic atrophy with intellectual disability (ID), and ataxia. Clinical features were recapitulated in a zebrafish knockdown model showing global developmental delay (DD), altered optic nerve morphology, and reduced forebrain. Bi-allelic variants in *SNF8* were documented to be associated with reduced ESCRT-II protein levels and defective autophagy, leading to the accumulation of autolysosomes and abnormal lysosomes in patient-derived fibroblasts.

## Material and methods

### Experimental design

Affected individuals with bi-allelic *SNF8* variants were collected via GeneMatcher^24^ and international collaborations. In-depth phenotyping of clinical symptoms, MRI findings, and examination of brain tissue was performed to compare the identified individuals and define the reported phenotypic spectrum. To provide further evidence of a disease association of variants in *SNF8*, functional studies in patient-derived fibroblasts as well as a zebrafish model were performed.

### Individuals and samples

Affected individuals were assessed at seven different clinical sites in Leipzig, Jerusalem, Rome, Bologna, and Ulm. Written informed consent for collection of tissue samples, clinical and genetic data, imaging studies, and functional studies was given by the legal guardians of all tested individuals. Approval of the local ethics committees was obtained, where necessary. Design and performance of the study was conducted according to the declaration of Helsinki. In some cases, connections between the respective institutes were made via the matchmaking platform GeneMatcher.^24^ Brain MRI images were acquired at each center and centrally evaluated by two board-certified neuroradiologists by consensus.

### Genetic studies

All genetic studies were approved by the local ethical committees of the respective institutes. For family A, exome sequencing (ES) (SureSelect Human All Exon V6, Agilent) of the index case (A1) and parents was performed in a diagnostic setting in Munich, Germany, as previously described.^25^ For individual A2, Sanger sequencing was performed to confirm the variants previously identified in the index case A1. For family B, ES (SureSelect All Human V6, Agilent) was performed in Leipzig, Germany. Genetic testing for family C was performed in Jerusalem, Israel using a SureSelect Human All Exon 50 Mb V5 Kit (Agilent). For family D, ES (TruSeq Rapid Exome, Illumina) was performed as part of a research project on hereditary optic neuropathies promoted by the Italian Ministry of Health at the IRCCS Istituto delle Scienze Neurologiche di Bologna, Bologna, Italy. Subjects E1 and E2 were analyzed in the context of a dedicated research project focused on undiagnosed individuals at the Ospedale Pediatrico Bambino Gesù, Rome, Italy. ES (SureSelect Human All Exon V6 or V7) was performed as previously reported.^26^^,^^27^ Individual F1 received trio ES in a research context using an Illumina Exome Panel enrichment Kit (45Mb size).

Methods used in ES data generation and processing, including variant filtering and prioritization are reported in the supplemental methods.

### Structural inspection of SNF8

The predicted alterations of SNF8 due to the identified *SNF8* variants were modeled using the crystal structure of the human ESCRT-II complex (PDB: 2ZME).^7^ Figures were generated using the Pymol software (https://pymol.org/2/).

### Analysis of brain MRI scans

In all individuals, structural brain MRI imaging included T1- and T2-weighted fast spin-echo scans. Two experienced neuroradiologists (L.L.G., B.Z.) retrospectively reviewed the available brain MRI scans.

In three affected individuals (individuals D1, E1, E2), volumetric 3D T1-weighted fast spoiled gradient echo (FSPGR) images (TR/TE/TI = 12.5/5.1/600 ms, 1 mm^3^ isotropic voxel) and in two (individuals D1, E1) DTI sequences (TR/TE = 10.000/87.5 ms, 25 diffusion gradient directions, b-value = 900 s/mm^2^, 1.25 mm in-plane reconstructed resolution, 4 mm slice thickness) were also available, permitting quantitative evaluation of white and gray matter volume and microstructural alterations.

In individuals D1, E1, and E2, MRI morphometric measurements of the anterior optic pathway were performed as previously reported^28^ and were compared with normal values reported in the literature for control subjects 12 to 18 years of age.^29^

Brain gray and white matter MRI volume analysis was performed with the software FreeSurfer (https://surfer.nmr.mgh.harvard.edu/). DTI imaging analysis was performed using the program DTIFIT (FMRIB Software Library v.6.0) to obtain MD maps. A processing pipeline developed in-house-generated histograms of MD for all pixels in the whole cerebral hemispheres and infratentorial compartment.^30^

Brain volumes and DTI values were compared with mean values of a population of healthy control subjects selected from the database of the Neuroimaging Laboratory (IRCCS Istituto delle Scienze Neurologiche di Bologna), designed to collect normative values of quantitative magnetic resonance parameters for clinical and research purposes.

### Neuropathological investigations of brain tissue from individual A2

The complete brain was prepared at autopsy and was fixed in formalin for a duration of two weeks before coronal slicing of 1 cm step size. Paraffin-embedded specimen sampled across the whole cerebrum, brain stem, cerebellum, and spinal cord were used for histological examination. Hematoxylin-eosin and luxol fast blue-periodic acid Schiff stains were carried out according to standard protocols. Immunohistochemistry (IHC) was performed on 5-μm-thick paraffin sections with an Autostainer Link 48 (Agilent/Dako) or a BenchMark GX (Roche) according to the instructions of the manufacturer using UltraView Kit (Roche) as detection system and diaminobenzidine as chromogen. Primary antibodies and their dilutions were LC3 (rabbit polyclonal, PM036, MBL; 1:1,000), neurofilament (mouse monoclonal, clone 2F11, M0762 Dako/Agilent; 1:500), HLA-DP, DQ, DR (mouse monoclonal, clone CR3/43, M0775 Dako/Agilent; 1:100), NeuN (mouse monoclonal, clone A60, MAB 377, Chemicon; 1:300), and GFAP (rabbit polyclonal, Z0334, Dako/Agilent; 1:2,000). An antigen retrieval was done for CR3/43 and NeuN by boiling in citrate buffer (pH 6) and for LC3 by boiling in Tris/EDTA buffer (pH 8). For IHC of LC3, the brain of an age-matched individual which died from a non-neurologic condition was used as control.

### Cell culture methods

Patient-derived primary fibroblasts (individual A2, D1, E1, and control cells from different subjects) cell lines as well as commercially available BJ primary fibroblast cells were maintained in DMEM medium-high glucose (D5786), supplemented with 10% fetal bovine serum (FBS), 100 U/mL penicillin, and 100 μg/mL streptomycin. All cells were cultured at 37°C supplemented with 5% CO_2_.

### Methods used for functional analysis of patient-derived fibroblasts

Patient-derived fibroblasts from affected individuals (A2, D1, and E1), control individuals, and commercially available BJ primary fibroblast cells were grown on coverslips to be analyzed by electron microscopy. After fixation, washing, and contrasting of cells, ultrathin sections of 100 nm were cut using an Ultracut UCT ultramicrotome (Leica, Austria). The prepared sections were subsequently analyzed by JEOL-JEM 1230 electron microscope at 80 kV. To identify the lysosomal compartment, cells were incubated with prepared colloidal gold protein conjugates (BSA-gold) for 24 h before being prepared for electron microscopy as mentioned above.

To study a possible accumulation of autolysosomes, cells derived from individual A2 or control individuals were left untreated or incubated with the Bafilomycin A1 (B1793 Sigma) to inhibit lysosomal acidification and then fixed and processed for immunofluorescence confocal microscopy with antibodies against LC3 (Rabbit anti-LC3, PM036, MBL; 1:400) and LAMP1 (Mouse anti-LAMP-1, AB 2296838, DHB; 1:200).

Additional details regarding the methods used, including a list of antibodies and reagents, can be found in the supplemental methods.

### RNA sequencing

RNA sequencing was performed from fibroblast RNA of individuals A2, E1, and D1 as previously described.^31^ RNA was isolated from the patient-derived skin fibroblasts with the RNeasy mini kit (Qiagen) according to the manufacturer’s protocol. RNA integrity number (RIN) was determined using the Agilent 2100 BioAnalyzer (RNA 6000 Nano Kit, Agilent). Strand-specific RNA-seq was performed according to the TruSeq Stranded mRNA Sample Prep LS Protocol (Illumina). Specifically, 1 μg of RNA was purified using poly-T oligo-attached magnetic beads and fragmented. RNA fragments were reverse transcribed with the First Strand Synthesis Act D mix. Second-strand cDNA was generated with Second Strand Marking Mix. The resulting double-stranded cDNA was subjected to end repair, A-tailing, adaptor ligation, and library enrichment. RNA libraries were sequenced as 100 bp paired-end runs on an Illumina HiSeq6000 platform. Reads from RNA-seq were demultiplexed and then mapped with STAR v.2.7.0a to the hg19 genome assembly, with default parameters plus setting the twopassMode to “Basic” to detect novel splice junctions. Aberrant expression was detected using the OUTRIDER pipeline.^32^ Z-scores are computed using the normalized counts. As controls for detection of aberrant expression and splicing, a set of 269 fibroblasts from individuals with Mendelian disorders as published before was used.^33^

### Proteomics

For individuals A2, E1, and D1 as well as the model cell line harboring the missense variant c.304G>A (p.Val102Ile) in a homozygous state, proteomics was performed at the BayBioMS core facility at the Technical University Munich, Freising, Germany. Fibroblast cell pellets containing 0.5 million cells were lysed in urea containing buffer and quantified using BCA Protein Assay Kit (Thermo Scientific). 15 μg of protein extract were further reduced and alkylated and the tryptic digest was performed using Trypsin Gold (Promega). Digests were acidified and desalted and TMT labeling was performed using TMT 10-plex labeling reagent (Thermo Fisher Scientific). Each TMT-batch consisted of 8 samples of affected individuals and 2 reference samples common to all batches to allow data normalization between batches. Each TMT 10-plex peptide mix was fractionated using trimodal mixed-mode chromatography. LC-MS measurements were conducted on a Fusion Lumos Tribrid mass spectrometer (Thermo Fisher Scientific), which was operated in data-dependent acquisition mode and multi-notch MS3 mode. Peptide identification was performed using MaxQuant v.1.6.3.4. Aberrant protein levels was detected using the PROTRIDER pipeline from the py_outrider package (https://github.com/gagneurlab/py_outrider). Z-scores are computed using the normalized counts. As controls for detection of aberrant expression and splicing, a set of 360 fibroblasts from individuals with Mendelian disorders from the in-house database of the Institute of Human Genetics in Munich were used.

### *In vivo* zebrafish experiments

Animal experiments were conducted under the approval of the Italian Ministry of Health (DGSA - 23/2019-PR). A detailed description of the methods used for the generation of the zebrafish model and the respective phenotype assessment can be found in the supplemental methods. In short, zebrafish NHGRI embryos were cultured following standard protocols.^34^ Morpholino (MO) targeting *snf8* ATG was injected in 1-cell stage fish to generate a transient LoF model, with or without co-injection of the human *SNF8* WT and mutant mRNAs. MO against *TP53* was also injected for all conditions to reduce general toxicity. All cloned sequences (human *SNF8*) and the absence of SNPs in the 24-region target by the MO in zebrafish were confirmed by Sanger sequencing.

### Phenotype assessment and brain and optic nerve analysis in zebrafish

Global phenotype penetrance was scored by bright-field microscopy at 24 and 48 hpf embryos. 48 hpf whole-mount fish specimens were fixed and double immunofluorescence was carried out assessing brain and optic nerve defects using antibodies against acetylated tubulin and phospho-histone 3 and imaged at confocal using an x,y,z scan mode, a 25× water immersion objective, 1.5 to 2 μm z-step size. The frequency of embryos with a range of brain (including ectopic proliferative cells) and ON phenotype from “normal” to “severe” was calculated and statistically compared. The ventral brain area and morphological parameters of the ON and OC were calculated using ImageJ*.*^35^

### Statistical analyses

Statistical analyses were performed using R 4.2.2 and RStudio 1.4. Visualization was done using the ggplot2 package.^36^ Statistical assessment of zebrafish morphological features was performed using Graphpad (Prism).

## Results

### Exome sequencing in nine individuals identifies bi-allelic variants in *SNF8*

Through international collaboration, we identified nine subjects from six unrelated families affected with a neurodevelopmental/neurodegenerative disorder who shared bi-allelic rare variants in *SNF8* (see Table 1). Although variable, the clinical phenotype of these individuals was consistent (see below), putatively linking the defective function of SNF8 to a single monogenic disease spectrum. Among these individuals, four subjects (A1, B1, C1, F1) had been analyzed using a trio-based ES approach mostly within the routine diagnostic setting (individuals A1, B1 and C1), while the others had been enrolled in research programs dedicated to undiagnosed individuals (proband-only ES, subjects D1, E1, E2). Additional details about ES performed can be found in the supplemental methods and Table S1. In cases with proband-only ES, the bi-allelic occurrence of the variants was confirmed by segregation analysis using Sanger sequencing (Figures S1–S3). Segregation analysis in individual C2 was performed by Sanger sequencing (data not shown).

**Table 1:** Clinical characterization of identified individuals with bi-allelic *SNF8* variants

**Individual**	**A1**	**A2**	**B1**	**C1**	**C2**	**D1**	**E1**	**E2**	**F1**
Pedigree	family A II-2	family A II-3	family B II-1	family C II-1	family C II-2	family D II-2	family E II-1	family E II-2	family F II-1

**Variants identified in *SNF8* (GenBank: NM_007241.4)**									

Variant 1	c.501C>A (p.Tyr167Ter)	c.501C>A (p.Tyr167Ter)	c.236C>T (p.Pro79Leu)	c.623G>T (p.Arg208Leu)	c.623G>T (p.Arg208Leu)	c.423−1G>C (p.?)	c.673_683delinsTGGA (p.Asp225TrpfsTer99)	c.673_683delinsTGGA (p.Asp225TrpfsTer99)	c.236C>T (p.Pro79Leu)
Variant 2	c.572G>A (p.Gly191Asp)	c.572G>A (p.Gly191Asp)	c.572G>A (p.Gly191Asp)	see above	see above	c.304G>A (p.Val102Ile)	c.304G>A (p.Val102Ile)	c.304G>A (p.Val102Ile)	c.304G>A (p.Val102Ile)
Zygosity	cmpd-het	cmpd-het	cmpd-het	hom	hom	cmpd-het	cmpd-het	cmpd-het	cmpd-het

**General information**									

Sex	female	female	male	male	female	male	male	male	female
Age at last visit	8 months	3 months	4.5 years	–	–	18 years	27 years	17 years	4 years
Age of onset	congenital	congenital	congenital	–	congenital	4 years	6 years	7 years	15 months
Age of death	8 months	3 months	alive	TOP	9 weeks	alive	alive	alive	alive

**Phenotype**									

NDD	yes (regression)	yes	yes	–	–	no	yes (mild)	yes (mild)	yes (mild)
ID	–	–	–	–	–	yes	yes	yes	yes
Seizures	yes	no	yes	–	no	no	no	no	no
Dysphagia	yes	yes (PEG)	yes (PEG)	–	yes (PEG)	no	no	no	no
Optic atrophy	N/A	N/A	N/A	–	no^g^	yes	yes	yes	no
Nystagmus	yes	N/A	yes	–	N/A	yes	yes	yes	no

**Brain MRI/ultrasound findings**									

Generalized brain atrophy	severe	severe	severe	–	severe	mild (total cerebral white matter volume reduction)	mild (total cerebral white matter volume reduction)	no	no
Abnormality of the CC	yes (hypoplasia)	yes (hypoplasia)	yes (aplasia)	yes (dysgenesia)	yes (aplasia)	no	no	no	yes (dysgenesia)
Abnormality of the cerebellum	no	no	no	–	yes (atrophy)	yes (cortex volume reduction)	yes (cortex volume reduction)	no	yes (cerebellar atrophy)
AOP volume reduction	no	no	no	–	no	yes	yes	yes	no

**Further investigations**									

EEG	generalized and multifocal epileptic discharges	normal	generalized and multifocal epileptic discharges	–	N/A	normal	normal	normal	normal
VEPs	N/A	N/A	N/A	–	N/A	pathologic	delayed latency and reduced amplitude	delayed latency and reduced amplitude	N/A

Overview of identified variants, general information, clinical symptoms, and results of diagnostic tests of the reported individuals with bi-allelic *SNF8* variants. Abbreviations: cmpd-het, compound-heterozygous; hom, homozygous; TOP, termination of pregnancy; NDD, neurodevelopmental delay; ID, intellectual disability; N/A, not available/not assessed; CC, corpus callosum; AOP, anterior optic pathway; VEPs, visual evoked potentials. "–" indicates individual was too young to determine the presence or absence of clinical features.

g bilateral hypoplastic optic nerves

**Figure 1:**
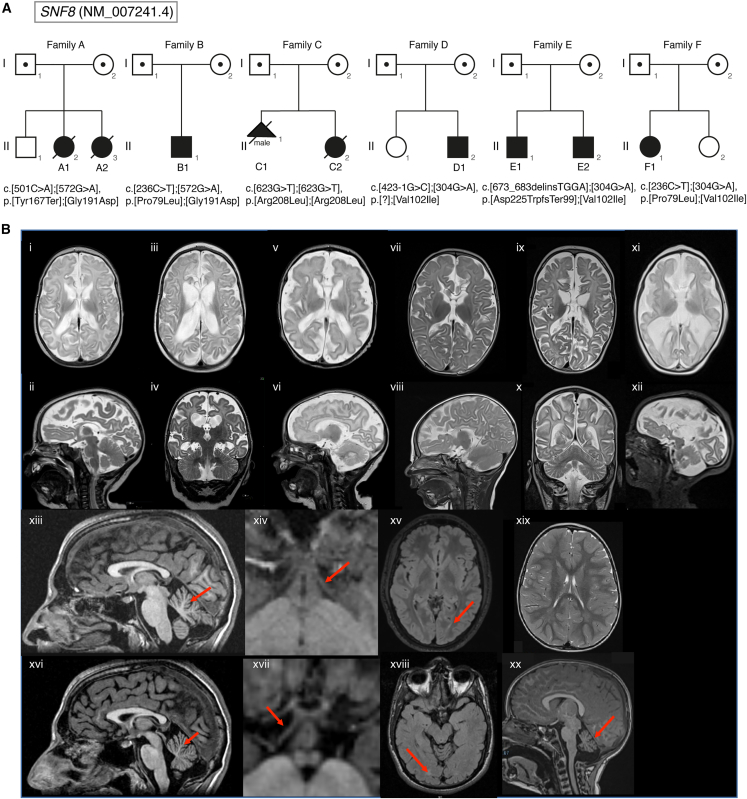
Pedigrees of the families with bi-allelic *SNF8* variants and T2-weighted brain MRI scans of affected individuals with bi-allelic variants in *SNF8* (A) Pedigrees of the families A–F. Variants identified in the individuals are depicted underneath the corresponding symbol. In family A, the healthy male sibling (II-1) of individuals A1 and A2 was not compound heterozygous for the *SNF8* variants. In family D, the healthy female sibling (II-1) was not compound heterozygous for the *SNF8* variants. Solid symbol, affected individual; circles, females; squares, males; slashed symbols, deceased. (B) Brain MRI scans of the affected individuals with pathogenic variants of *SNF8*. (i–xii) T2-scans of the severely affected individuals. Images of A1 at the age of two (i, ii) and six months (iii, iv), of subject A2 at the age of two months (v, vi), of subject B1 at the age of four (vii, viii) and 15 months (ix, x), and of subject C2 at the age of one month (xi, xii). All severely affected subjects (i–xii) show a pronounced and persistent white matter hyperintensity, an atrophy of the cerebrum and callosal hypoplasia. The brainstem appears comparatively normal and the cerebellum is less severely affected. Pachygyria was noted in individual A2 (v). Note the rapidly progressive enlargement of the lateral ventricles due to cerebral white matter loss in subjects A1 and B. (xiii–xx) MRI images of the less severely affected individuals. Individuals D1 (xiii–xv) and E1 (xvi–xviii), both recorded at the age of 16 years, 3D volumetric T1 showing cerebellar atrophy (xiii and xvi, red arrows), as well as severe volume reduction of the anterior optic pathway including optic nerves, optic chiasm, and optic tracts (xiv and xvii, axial magnification of the 3D volumetric T1, red arrows). Moreover, on axial T2 (xv) or FLAIR (xviii) images, slight hyperintensity of the parieto-occipital white matter was observed (red arrows); notably automatic software evaluation (freesurfer) demonstrated a total cerebral white matter volume reduction in both affected individuals. (xix and xx) MRI scan of individual F1 at the age of two years showing normal myelination and white matter volume in T2 (xix) and a slightly dysmorphic callosal body as well as slight cerebellar atrophy in a sagittal T1 scan (xx, red arrow).

In all subjects, analysis of genetic data excluded the occurrence of other (likely) pathogenic variants compatible with known Mendelian disorders based on the expected inheritance model, clinical presentation, and variant annotation information. In all families, the healthy parents were heterozygous for the variants identified in the probands, and genotyping of the additional family members confirmed co-segregation of the disease with bi-allelic occurrence of the identified *SNF8* (GenBank: NM_007241.4) variants (Figures 1 and S1–S3). All variants except c.501C>A (p.Tyr167Ter), c.304G>A (p.Val102Ile), c.236C>T (p.Pro79Leu), and c.572G>A (p.Gly191Asp) are not listed in gnomAD v.4.0.0 with the latter two representing rare variants with very low allele frequencies there (see Table S2).^16^ The variant encoding p.Val102Ile is present in a homozygous state in one apparently healthy individual from the gnomAD population. Among the seven variants, three were predicted to affect transcript processing (c.423−1G>C [p.?]) or result in a truncated protein (p.Tyr167Ter) or in a protein with a divergent C-terminal portion (c.673_683delinsTGGA [p.Asp225TrpfsTer99]), while the rest were missense. Of note, three of the four missense changes (p.Pro79Leu, p.Val102Ile, and p.Gly191Asp) were recurrent and were predicted to have a damaging impact on protein function, further strengthening the hypothesis of their clinical relevance. Table S2 lists all variants, their frequency in gnomAD (v.4.0.0), and their *in silico* metrics.

### Phenotypic spectrum of affected individuals ranges from lethal leukoencephalopathy to syndromic optic atrophy

Table 1 summarizes the clinical phenotype of the reported individuals, comprising four females (siblings A1 and A2 [family A II-2 and II-3], individuals C2 [family C II-2], and F1 [family F II-1]) and four males (individuals B1 [family B II-1], D1 [family D II-2], and siblings E1 and E2 [family E II-1 and II-2]) (Figure 1) from the six unrelated families. In family C, a pregnancy of a male fetus (II-1 in Figure 1) was terminated at 25 weeks due to brain malformations identified prenatally. Overall, four individuals (A1, A2, B1, C2) showed a severe developmental and epileptic encephalopathy with leukoencephalopathy and early death in three of those cases. Two individuals died too young to develop epilepsy. In contrast, three individuals (D1, E1, E2) presented with a milder phenotype comprising ID and degenerative involvement of the optic nerve. Finally, a clinical picture of congenital ataxia was observed in the 4-year-old subject F1. Case reports of each individual can be found in the supplemental note.

Aside from the reported pregnancy termination, all eight individuals were delivered at term. Postnatal adaptation was normal in 6 out of 8 individuals, while individuals A2 and C2 showed repeated drops in oxygen saturation. All four children from the severely affected group showed a global developmental disorder with no achievement of major developmental milestones and developed secondary microcephaly. Muscular hypotonia was present in all severely affected individuals, with one subject developing a spastic movement disorder. In individuals A1 and B1, a pathologic EEG pattern with multifocal and generalized epileptic discharges was recognized (see Figure S4 for exemplary EEGs of individual B1). Individual A1 developed myoclonic seizures and soon after tonic seizures with ictal electrodecrements on EEG. EEG abnormalities and seizures were absent in individuals A2, C2 and F1. Individual C2 was reported to have bilateral hypoplastic optic nerves; the young age hampers the assessment of a possible degenerative pathology of the optic nerves. Neonatal feeding difficulties and dysphagia were an additional unifying feature, three individuals required gastric tube feeding. Three affected individuals died in the first year of life in a palliative care setting: individual A1 died due to cardiac arrest during status epilepticus, while individuals A2 and C2 both passed away due to a respiratory infection possibly caused by dysphagia. Individual B1 is almost 3 years old, severely disabled and taken care of by a palliative care team.

For the four remaining individuals presenting with a milder phenotype, mild DD/ID, mainly affecting speech and language development, was noted. Optic (hypo)-atrophy as confirmed by optical coherence tomography, with an age of onset ranging from 4 to 7 years, was reported in three individuals (D1, E1, E2), with siblings E1 and E2 having abnormal visually evoked potentials with delayed latency and reduced amplitude. Besides congenital ataxia in individual F1, neurological phenotypes including seizures were absent in this group. Individuals D1, E1, and E2 survived into adulthood; subject F1 is currently 4 years old. Of note, all four individuals with a milder phenotype shared the p.Val102Ile missense variant. Eye movement abnormalities were a unifying feature for both mild and severely affected individuals with individuals A1, B1, D1, E1, and E2 presenting with nystagmus. Variable congenital anomalies of minor severity were detected including ureterocele in individual A1, undescended testes in individual B1, and limb contractures in individual C2.

Additionally, the in-house exome database of the Institute of Human Genetics in Munich currently containing about 27,500 exome datasets was queried for individuals harboring bi-allelic variants in *SNF8*. This yielded an additional unrelated individual harboring the missense variant predicting the p.Val102Ile substitution at homozygous state. This individual had an alternative diagnosis (i.e., mitochondrial complex III deficiency, nuclear type 1 [MIM: 124000]) caused by a pathogenic homozygous variant in *BCS1L* (GenBank: NM_004328.5; c.232A>G [p.Ser78Gly]). The postnatal presentation of seizures, liver failure, and muscular hypotonia could be explained by the confirmed mitochondrial disorder. Of note, the short survival of this subject hampered a long-term assessment to investigate the possible occurrence of ID and optic atrophy due to the homozygous *SNF8* variant later in life. Additional phenotypic data available are presented in Table S3.

### Brain MRI studies reflect the broad clinical spectrum associated with variants in *SNF8*

Brain MRI imaging of individuals A1 (age of two and six months), A2 (age of two months), B1 (age of four and fifteen months), and C2 (age of one month) showed pronounced and progressive white matter atrophy of the cerebrum as well as hypo- or aplasia of the corpus callosum beginning at a very early age (Figure 1). Individuals D1 (age of 16 years), E1 (age of 16 years), and E2 presented with a severe volume reduction of the intracranial anterior optic pathway including optic nerves (ON), optic chiasma (OC), and optic tracts (2 SD below normal values). In individual D1 brain MRI volume analysis showed a reduction of the total cerebral white matter volume (−17% bilaterally) and cerebellar cortex volume (−13% left and −21% right hemisphere). Brain diffusion tensor imaging (DTI) analysis showed increased mean diffusivity (MD) in the cerebellar hemispheres (+13%, bilaterally). In individual E1, brain MRI volume analysis showed total white matter volume reduction (−20% bilaterally), and the temporal cortex (−17% bilaterally), cerebellar cortex (−19% on the left and −17% on the right), as well as putamen (−18% left and −17% right) had volume reduction. Brain DTI analysis showed increased MD in the cerebellar hemispheres (+9% left and +8% right). In contrast, brain MRI of individual F1 at the age of two years revealed cerebellar atrophy and a slightly dysmorphic corpus callosum. The anterior optic tract appeared normal, but note that the MRI scan was performed at an earlier age than that in individuals E1 and E2.

### Neuropathological investigations of brain tissue show severe white matter disease

**Figure 2:**
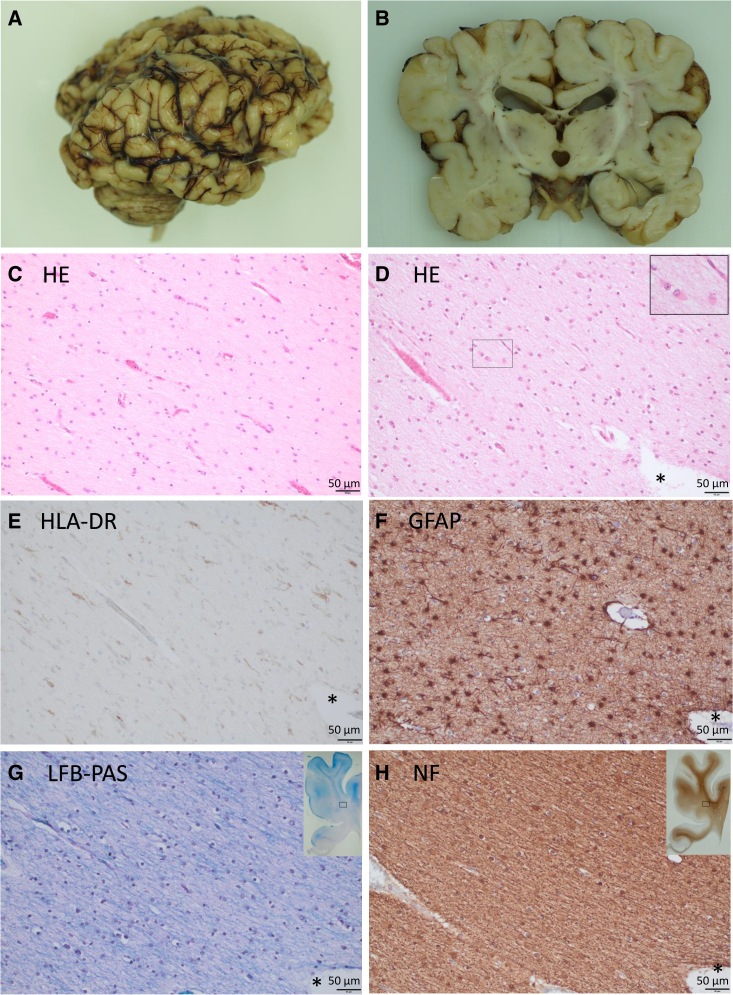
Macroscopic and microscopic alterations in individual A2 deceased at the age of 3 months (A) Lateral view on the formalin-fixed autopsy brain. (B) Coronally cut slice of the formalin-fixed brain at the level of thalamus/hippocampus showing a massive reduction of the subcortical white matter and a severe atrophy of the corpus callosum. The gyri appear coarse. (C–H) Frontal subcortical white matter at level of anterior cingulate gyrus; (C) age-matched control. (D–H) Consecutive sections in individual A2. (C and D) Hematoxylin-eosin (HE) stains. Compared to the age-matched control case (C), the astrocytes of individual A2 (D) are reactively altered showing enlarged eosinophilic cell bodies (inset displays zoomed marked area). The number of astrocytes is also increased in individual A2. (E) With an antibody against human leukocyte antigen-DR isotype (HLA-DR), numerous activated microglial cells (brown color) are visualized as a sign of a resorptive process. (F) The immunohistochemical detection of glial fibrillary acidic protein (GFAP; brown color) illustrates the large number of reactively changed astrocytes. (G) Myelin is dramatically reduced (blue color) in a luxol fast blue-periodic acid Schiff (LFB-PAS) stain, while in (H) the axons are relatively well preserved as demonstrated by immunohistochemistry for neurofilament (NF; brown color). Insets in (G) and (H) show overviews of the slides, rectangles mark zoomed areas. ^∗^Highlights the same blood vessel in consecutive tissue sections. Scale bars in (C)–(H) correspond to 50 μm.

To investigate the observed defects further, neuropathological examination of brain and spinal cord tissue, which was preserved during the autopsy of individual A2 following death at the age of three months, was performed. Macroscopic findings of the cerebrum reflected MRI findings (Figures 2A and 2B) revealing pachygyria, enlarged sulci, and reduced cerebral white matter in coronal sections. The corpus callosum was thinned, measuring 2 mm.^37^ Reduced white matter was also apparent—although less severe—in the cerebellum and the spinal cord. Microscopically, cerebral tissue samples showed a marked loss of myelin leading to a reduction of white matter substance, reactive gliosis with increased numbers of reactive astrocytes (Figures 2D–2H and Figures S5–S7), as well as microglia activation (Figures 2E, S6, and S7). Cortex architecture was normal. Apart from the described brain alterations, there were no relevant organ abnormalities or congenital malformations identified during the autopsy.

### Functional impact of the identified missense variants on SNF8

In total, three putative LoF variants and four missense variants were identified. The four missense variants uniformly affected evolutionary highly conserved amino acid residues (Figure S8). *In silico* prediction scores for three of the four missense variants (p.Gly191Asp, p.Pro79Leu, and p.Arg208Leu) consistently supported a high impact of the respective amino acid substitutions on protein function (Combined Annotation Dependent Depletion [CADD] score ranging between 27.3 to 33.0,^38^ Rare Exome Variant Ensemble Learner [REVEL] scores ranging from 0.683 to 0.953^39^). In contrast, prediction tools suggested a less disruptive impact of the p.Val102Ile amino acid substitution. In line with a likely damaging effect of the identified missense changes, however, all variants affected residues that were spotted in regions documented to be intolerant to variation as observed in data obtained from MetaDome (Figure S9).^40^

**Figure 3:**
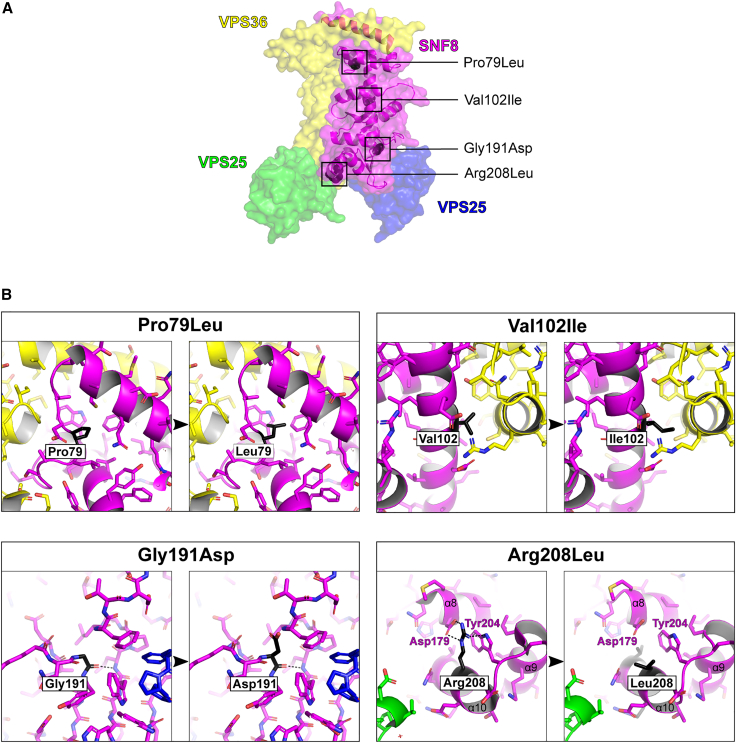
Representation of the 3D structure of SNF8 with the observed missense variants (A) The surface rendering of human ESCRT II complex (PDB: 2ZME^7^). VPS36 is yellow, SNF8 is magenta, and 2 molecules of VPS25 are green and blue. The ribbon diagram with secondary structure elements is shown for SNF8, and the residues affected by the reported missense variants are shown as black spheres. (B) Magnified view of the residues affected by the reported missense variants. The affected residues are shown in black and labeled accordingly. Hydrogen bonds involving affected residues are depicted as dashed black lines. The variants p.Pro79Leu and p.Gly191Asp introduce larger residues that lead to steric clashes with surrounding residues and likely affect the conformation and stability of SNF8. In contrast, the p.Arg208Leu variant replaces the long and charged arginine with a short and hydrophobic leucine, resulting in a loss of interaction with aspartic acid 179 and tryptophan 204, thus most likely also affecting the conformation and stability of SNF8. The most conservative amino acid substitution with the least impact is p.Val102Ile, in which the small hydrophobic valine is replaced by a slightly larger (one atom) hydrophobic isoleucine.

None of the affected residues is involved in direct binding of other subunits of ESCRT-II (Figure 3). However, protein modeling of the identified missense variants suggested a conformational change in the aberrant SNF8 that could affect interactions with VPS36 or VPS25 or the overall stability of the protein (Figure 3). Residues Pro79 and Val102 are indeed located near the interface between SNF8 and VPS36, and the missense variants p.Pro79Leu and p.Val102Ile were predicted to alter SNF8 stability and affinity between these two subunits. Similarly, residue Gly191 is located near the interface between SNF8 and VPS25. The replacement of the small, conserved Gly with a larger, positively charged residue (Asp) was expected to lead to steric clashes that most likely affect the conformation and stability of SNF8 and its interactions with VPS25. Finally, Arg208 stabilizes the 3-helix bundle within SNF8 through conserved electrostatic interactions with Asp179 and π-π interactions with Trp204. Loss of this interaction would most likely compromise stability of SNF8.

### Transcriptomics and proteomics from patient-derived fibroblasts show loss of ESCRT-II complex with genotype-phenotype correlation

To further investigate the effects of the identified variants in *SNF8*, fibroblasts from a skin biopsy of individuals A2, D1, and E1 as well as the unrelated individual harboring the variant encoding p.Val102Ile in a homozygous state were cultured, and RNA sequencing and proteomic analyses were performed. For individual A2, RNA sequencing showed an overall decreased expression of *SNF8* with a mostly monoallelic expression of the A allele affecting the identified *SNF8* missense variant p.Gly191Asp (variant allele fraction 93%) (Figure S10). This was also observed for individual D1, where mostly monoallelic expression of the A allele affecting the missense variant p.Val102Ile was documented in fibroblasts (variant allele fraction 90%) (Figure S11). Unbiased analysis of the three samples directed to identify expression outliers, monoallelic expression, and aberrant splicing did not reveal any additional findings hinting an alternative diagnosis.

**Figure 4:**
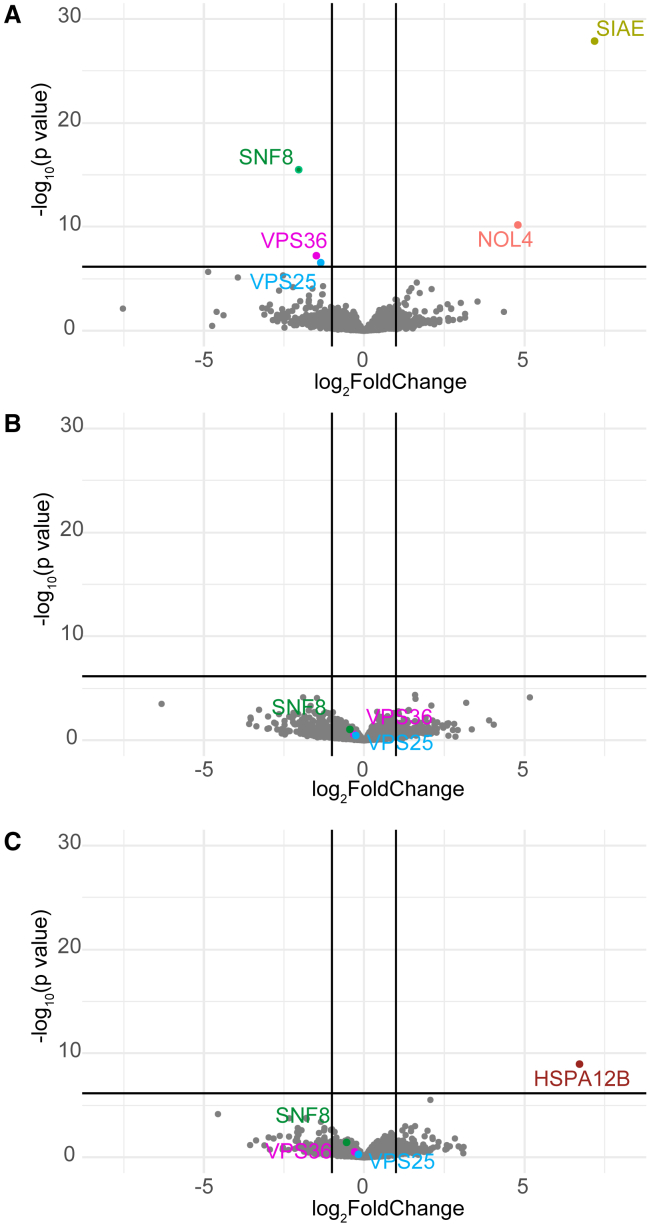
Effects of bi-allelic variants in *SNF8* on SNF8 levels and ESCRT II complex subunits Volcano plot of the proteomics analysis performed on cultured fibroblasts from individual A2 (A), D1 (B), and E1 (C). SNF8, VPS25, VPS36, and proteins with statistically different levels are highlighted in color. Vertical black lines indicate log2fold changes of −1 and 1. Horizontal black lines depict significance level of p = 7.14 × 10^−6^ (Bonferroni correction for 7,000 hypotheses representing the number of proteins identified). Note the significant reduced protein levels of ESCRT II complex subunits SNF8, VPS25, and VPS36 for individual A2.

By performing proteomic analysis, we observed that SNF8 as well as the two physically interacting subunits of ESCRT-II, VPS36, and VPS25 were significantly decreased in cells from individual A2 (severe phenotype) (SNF8: fold change [fc] = 0.25, adjusted p value p_adj_ = 7.8E−15; VPS36: fc = 0.39, p_adj_ = 0.003; VPS25: fc = 0.37, p_adj_ = 0.005) compared to controls (Figure 4A). In contrast, a reduction of SNF8 levels that however did not reach statistical significance was observed in fibroblasts from individuals D1 and E1 (milder phenotype) (D1: SNF8: fc = 0.74, E1: SNF8: fc = 0.68) (Figures 4B and 4C). In these samples, the rank of protein level intensity were 19 and 14 out of 323 internal control samples, respectively (Figure S12). Additionally, in these individuals there was no significant reduction of VPS36 and VPS25 protein levels, conversely to the reduction identified in A2 (D1: VPS36: fc = 0.81; VPS25: fc = 0.84; E1: VPS36: fc = 0.81; VPS25: fc = 0.87). Apart from SNF8 and ESCRT-II subunits, there were no consistent outliers identified in the three investigated fibroblast samples (for outliers per sample see Figure 4).

To model the effect of the hypomorphic missense variant p.Val102Ile, we took advantage of a fibroblast cell line from the individual harboring the missense variant in a homozygous state. Proteomics analysis did not show a significant reduction of ESCRT-II proteins; consistently, a slightly higher SNF8 level compared to those documented in primary fibroblasts from individuals A2, D1, and E1 was observed (SNF8: fc = 0.79; Figures S12 and S13); SNF8 level intensity rank was 23 out of 323 controls without reduction of the additional ESCRT-II subunits (VPS36: fc = 0.91; VPS25: fc = 0.82).

### Evidence of impaired autophagy in primary fibroblasts and brain tissue of an affected individual

**Figure 5:**
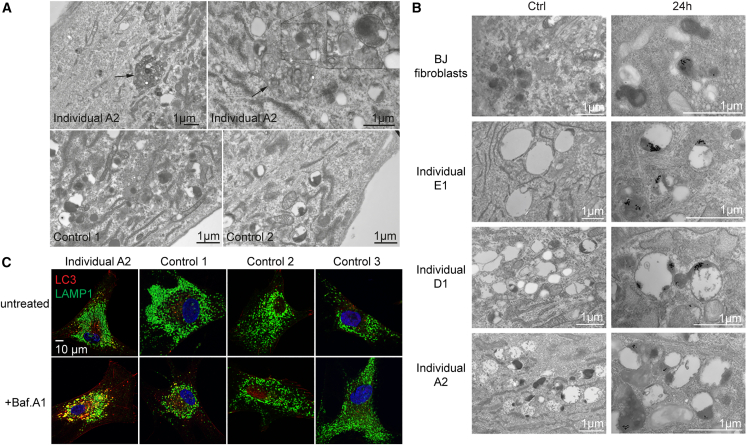
Bi-allelic variants in *SNF8* in individual A2 lead to loss of ESCRT II complex resulting in an autophagic phenotype in fibroblasts (A) Fibroblasts derived from individual A2 (top) or control individuals (bottom) were analyzed by transmission electron microscopy. Arrows indicate large vesicular structures that contain cytoplasmic content, consistent with being autolysosomes. Scale bars: 1 μm. (B) Fibroblasts were incubated with BSA-gold (10 nm) for 24 h and washed and then the gold was chased overnight to accumulate in lysosomal compartments. Cells were then studied by transmission electron microscopy. Images of untreated fibroblasts are found on the left (Ctrl) whereas images on the right side (24 h) represent fibroblasts stained with BSA-gold. In BJ control fibroblasts, most of the gold tracer is found in uniformly dense lysosomes. In patient-derived cells, the gold is found in the dense part of bigger lysosomal compartments, which mostly consist of an electron-lucent lumen. This phenotype is similar for the various patient-derived fibroblasts. Scale bars: 1 μm. (C) Fibroblasts derived from individual A2 or control individuals were left untreated of incubated with Bafilomycin A1 and then fixed and processed for immunofluorescence confocal microscopy with antibodies against LC3 and LAMP1. Co-localization between the two markers after Bafilomycin A1 treatment is indicative of autolysosomes. Scale bar: 10 μm.

Given the known role of ESCRT in trafficking and autophagy,^8^^,^^11^ we next examined ligand-induced EGFR degradation and autophagic flux in cultured fibroblasts of individual A2. EGFR degradation was not detectably impaired in patient-derived fibroblasts as compared to fibroblasts from control individuals and standard HeLa cells, although variability of EGFR levels between cell lines was high (Figure S14). Electron microscopy of patient-derived fibroblasts showed an accumulation of enlarged vesicular structures that contained cytoplasmic material, indicating that autolysosomes, the fusion products of autophagosomes and lysosomes, accumulate in these cells (Figure 5A, arrows). In addition, lysosomes, identified by accumulation of internalized BSA-gold, had an aberrant morphology in patient-derived fibroblasts, with enlarged size and a largely electron lucent lumen (Figure 5B). To functionally address the autophagic flux, we next treated cells with bafilomycin A1, which inhibits lysosomal acidification and protein degradation and the autophagosome-lysosome fusion. We used antibodies against LC3 and LAMP1 to visualize autophagic membranes and lysosomes by confocal microscopy. Structures positive for both LC3 and LAMP1 identify autolysosomes.^41^ As shown in Figure 5C, whereas LC3- and LAMP1-positive structures could be identified in both patient-derived and control fibroblasts, an increased number of vesicles showing co-localization between the two markers was evident in patient-derived fibroblasts treated with bafilomycin A1 compared to the untreated condition. This analysis confirms an accumulation of autolysosomes in the patient-derived fibroblasts. Taken together, the electron and confocal microscopy analyses indicate that autolysosomes and aberrant lysosomes accumulate in patient-derived fibroblasts and that proper autophagic flux is inhibited in these cells.

Since neurologic manifestations were the most prominent phenotypic feature in the reported individuals, we also asked whether we could confirm impaired autophagy as a possible underlying disease mechanism in brain tissue of individual A2 by immune-detection of the autophagy-related protein LC3. Immunostaining of LC3 in frontal lobe sections revealed strong staining in cells of the internal pyramidal cell layer as well as reactive astrocytes of the white matter. In contrast, age-matched control samples showed only weak staining in neurons and glia cells (Figure S15). In addition, cerebellar tissue sections showed strong LC3 staining in the outer granular cell layer of the developing cerebellar cortex and in neurons in the white matter substance, while the staining was weaker and occurred in fewer cells of the control samples. When observing the staining pattern with a higher magnification, cytosolic staining as well as staining of vesicular structures and the nuclear membrane were visible (Figure S16). Thus, findings in the brain tissue were consistent with the accumulation of autolysosomes observed in fibroblasts *in vitro*.

### Snf8 LoF in zebrafish embryos causes a developmental phenotype that is rescued by wild-type SNF8 but not by the disease-associated variants

**Figure 6:**
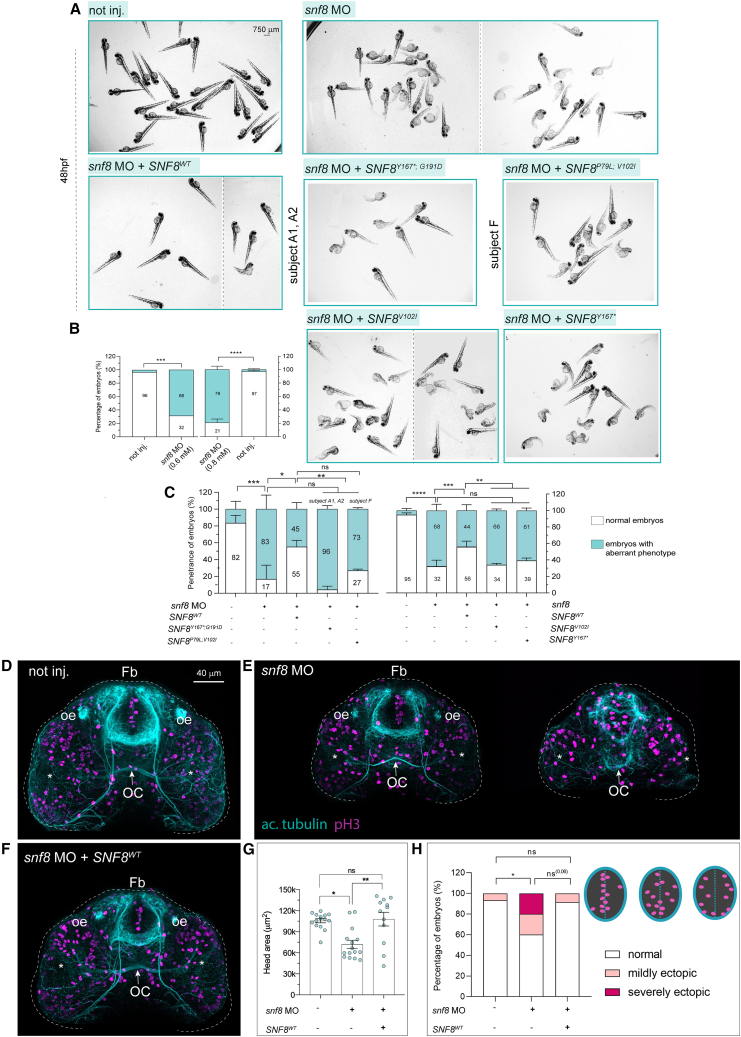
Snf8 loss of function in zebrafish leads to developmental defects that are rescued by expression of the wild-type *SNF8* cDNA but not disease-associated variants, and causes reduced anterior brain area and ectopic proliferation (A) Representative bright-field pictures of fish not injected (not inj.), zebrafish injected with *snf8* MO with or without mRNA encoding wild-type SNF8, or the disease-associated *SNF8*^Y167∗;G191D^, *SNF8*^P79L;V102I^, *SNF8*^V102I^, or *SNF8*^Y167∗^ alleles at 48 hpf. The dashed vertical lines separate different images from different representative fields of view. (B and C) Quantification of the percentage of fish showing normal (white) or aberrant (cyan) development upon injection of different *snf8* MO doses (B) and of *snf8* MO at 0.8 mM with or without the WT and mutant *SNF8* mRNAs (C). In (B), n = 75 (not inj. on the left), 19 (*snf8* MO 0.6 mM), 67 (*snf8* MO 0.8 mM), and 104 (not inj. on the right). In (C) (left), n = 39 (not inj.), 8 (*snf8* MO at 0.8 mM), 23 (*snf8* MO + *SNF8*^WT^), 11 (*snf8* MO + *SNF8*^Y167∗;G191D^), 18 (*snf8* MO + *SNF8*^P79L;V102I^). In (C) (right), n = 165 (not inj.), 99 (*snf8* MO at 0.8 mM), 98 (*snf8* MO + *SNF8*^WT^), 55 (*snf8* MO + *SNF8*^V102I^), 39 (*snf8* MO + *SNF8*^Y167∗^). (D–F) Representative confocal maximum intensity projections of fish fluorescently stained with antibodies against acetylated tubulin (cyan) and pH3 (magenta) from not injected (not inj.) (D), mild and severe cases of fish injected with *snf8* MO (E), and fish co-injected with *snf8* MO (at 0.8 mM) and *SNF8*^WT^ (F). Fish injected with *snf8* MO show a reduced anterior brain area and ectopic proliferative cells within the forebrain partially rescued by *SNF8*^WT^. (G) Quantification of the overall brain area from the confocal z-projections. (H) Number of embryos showing “normal” or “mildly or severely ectopic” proliferative cells (pH3+) within the anterior ventral forebrain. n = 15 (not inj. and *snf8* MO) and 12 (*snf8* MO + *SNF8*^WT^). Data are expressed as boxplot, for experiments including different batches mean ± SEM are shown. Number of replicates in (B), one and two (left and right graphs, respectively). In (C) (left), number of replicates: three for not inj, *snf8* MO, *snf8* MO + *SNF8*^WT^, and *snf8* MO + *SNF8*^Y167∗;G191D^; two for *snf8* MO + *SNF8*^P79L;V102I^. In (C) (right), number of replicates: six (not inj, *snf8* MO, and *snf8* MO + *SNF8*^WT^); three (the other two mutants). Two-sided chi-square test is used to assess statistical significance in a 2×2 contingency table. In (B), not inj. vs*. snf8* MO, ^∗∗∗^p = 0.0008 left and ^∗∗∗∗^p < 0.0001 right. In (C) (left), not inj. vs. *snf8* MO, ^∗∗∗^p = 0.0005; *snf8* MO vs. *snf8* MO + *SNF8*^WT^, ^∗^p = 0.0312; *snf8* MO + *SNF8*^WT^ vs. + *SNF8*^Y167∗;G191D^, ^∗∗^p = 0.0086; *snf8* MO + *SNF8*^WT^ vs. *snf8* MO + *SNF8*^P79L;V102I^, not significant (ns, p = 0.06); *snf8* MO vs. mutants, ns. In (C) (right): not inj. vs*.* MO, ^∗∗∗∗^p < 0.0001; *snf8* MO vs*. snf8* MO + *SNF8*^WT^, ^∗∗∗^p = 0.0008; *snf8* MO + *SNF8*^WT^ vs*. snf8* MO + *SNF8*^V102I^, ^∗∗^p = 0.0021; *snf8* MO + *SNF8*^WT^ vs*. snf8* MO + *SNF8*^Y167∗^, ^∗∗^p = 0.0022 (ns: not significant). In (G) non-parametric Kruskal-Wallis test with Dunn’s *post hoc* test is used for statistical assessment (not inj. vs*. snf8* MO, ^∗^p = 0.0141; *snf8* MO vs*. snf8* MO + *SNF8*^WT^, ^∗∗^p = 0.0032; ns = not significant). In (H), two-sided chi-square test is used: not inj. vs*. snf8* MO, ^∗^p = 0.0309; not inj. vs*. snf8* MO + *SNF8*^WT^, p = 0.8695 (ns, not significant); and *snf8* MO vs*. snf8* MO + *SNF8*^WT^, p = 0.0621 (ns). Fb, forebrain; oe, olfactory epithelium; OC, optic chiasm indicated by a white arrow. Asterisks indicate the eyes.

Next, to investigate the consequences of SNF8 LoF during embryonic development, we generated a zebrafish *snf8* knockdown model. The zebrafish genome encodes a single protein ortholog to human SNF8. The two proteins showed a high amino acid conservation, including the residues that were affected by the identified variants (Figure S8). We silenced *snf8* expression in zebrafish embryos using an ATG morpholino (MO)-mediated approach and evaluated the phenotype occurring in morphant fish and fish co-injected with the *snf8* targeting-MO oligonucleotide and mRNA encoding WT SNF8. To minimize off-target effects, we injected one-cell stage embryos deriving from a fish population showing no SNPs in the 5′ UTR region targeted by the MO oligonucleotide (Figure S17) and co-injected an equal amount of MO against *TP53*, as reported.^42^ Given the clinical manifestation of the disease (i.e., DD, variable involvement of the brain with cortical or cerebellar atrophy and/or optic nerve atrophy, Figure 1; Table 1), we scored the developmental progression in 24 hpf and 48 hpf embryos and examined brain and ON morphology. Compared to not-injected siblings, a statistically significant fraction of embryos injected with the *snf8* MO exhibited a stunted appearance, with evident DD, curly tail phenotype, reduced pigmentation, and small head/eyes of variable severity (Figures 6A and S18). The incidence of this phenotype was dependent upon the injected dose of MO and a statistically significant partial rescue was obtained by co-injecting the mRNA encoding WT SNF8 (Figures 6B and 6C).

To validate the pathogenicity and the variable functional impact of the disease-associated variants, we co-injected *snf8* MO with *SNF8* mRNA harboring variant combinations associated with the severe clinical phenotype occurring in subjects A1 and A2 (p.Tyr167Ter and p.Gly191Asp) and the milder features characterizing subject F (p.Pro79Leu and p.Val102Ile). In both cases, the coexpressed variants failed to significantly rescue the global embryo phenotype, corroborating their pathogenicity. Moreover, a more severe phenotypic impact in embryos coexpressing the *SNF8* alleles encoding p.Tyr167Ter and p.Gly191Asp compared to those microinjected with alleles encoding p.Pro79Leu and p.Val102Ile was observed (approximately 95% vs. 70% aberrant embryos) (Figures 6A and 6C), in line with the clinical findings. Accordingly, we noted that variants’ combination from subjects A1 and A2 already failed to rescue early embryo lethality observed in *snf8* morphant fish (mean ± SD: 79% ± 12,8%, for *snf8* MO + *SNF8*^Y167∗;G191D^ compared to 78.6% ± 12.4% for *snf8* MO, n = 18 and 11, respectively). Reduced lethality was observed for subjects F variants’ combination co-injected with *snf8* MO (mean ± SD: 59.2% ± 1.8% for *snf8* MO + *SNF8*^P79L;V102I^ compared to 78.6% ± 12.4% for *snf8* MO, n = 21 and 11, respectively). Injection of *SNF8*^Y167∗^ and *SNF8*^V102I^ variants alone also failed to rescue the snf8 knockdown phenotype in fish, further showing the pathogenicity of the single variants (Figures 6A and 6C). Overall, the data therefore confirmed the pathogenicity of the tested *SNF8* variants, and for a subset of those corroborated their differential impact, which is consistent with their associated variable clinical spectrum of the disease.

### Snf8 morphant fish exhibit a reduced brain size and aberrant optic nerve and optic chiasm morphology

To investigate more in detail whether snf8 LoF could recapitulate in fish the specific brain phenotypes observed in affected individuals, we performed immunofluorescence analysis on the morphant fish and morphants expressing WT *SNF8* by staining the overall axonal scaffold (acetylated tubulin) and proliferating precursors (phospho-H3 [pH3]). Morphometric analysis on confocal *x,y,z* acquisitions of the ventral brain showed variable phenotypes among morphant fish, with a reduction of the brain area (Figures 6D and 6E), which was significantly rescued in the population of fish co-injected with WT *SNF8* (Figures 6F and 6G).

Moreover, by inspecting pH3+ precursor cells within the forebrain proliferative zone of 48 hpf fish in morphant individuals, we noted the occurrence of ectopic cells, sparsely populating the region away from the ventricle where they are normally found. Again, this feature was partially rescued in fish expressing WT *SNF8* (Figure 6H).

**Figure 7:**
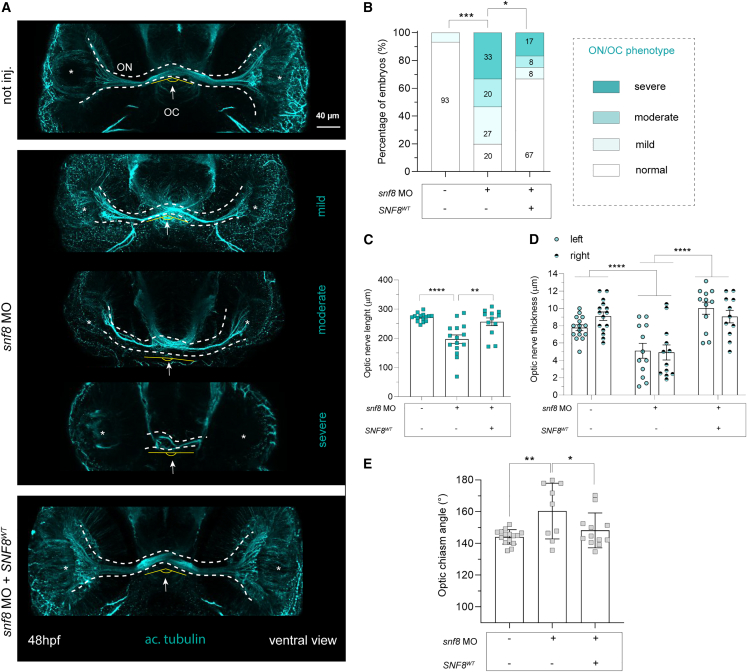
Zebrafish embryos injected with *snf8* MO exhibit altered optic nerve and optic chiasm morphology at 48 hpf partially rescued by *SNF8*^WT^ (A) Representative confocal maximum intensity projections of fish fluorescently stained with the antibody against acetylated tubulin (cyan) from not injected (not inj., up), mild, moderate, and severe cases of fish injected with *snf8* MO (center) and fish co-injected with *snf8* MO and mRNA encoding SNF8^WT^ (bottom). The white asterisks indicate the position of the eye. (B) Quantification of the percentage of fish showing normal (white) or mild, moderate, or severely aberrant (gradients of cyan) ON phenotypes as described in the main text. n = 15 (not inj.), 15 (*snf8* MO), and 12 (*snf8* MO + *SNF8*^WT^). Two-tailed chi-squared test is used to assess statistical significance of the occurrence of the phenotype (moderate + severe) in a 2×2 contingency table: not inj. vs*. snf8* MO, ^∗∗∗^p < 0.0001; *snf8* MO vs*. snf8* MO + *SNF8*^WT^, ^∗^p = 0.0330. (C and D) Quantification of the optic nerve length, measured as ON extension between two eyes and thickness, measured on both side of ON. In (C), n = 15 (not inj.), 15 (*snf8* MO), and 12 (*snf8* MO + *SNF8*^WT^). One-way ANOVA with Tukey’s *post hoc* test is used to assess statistical significance: not inj. vs*. snf8* MO, ^∗∗∗∗^p < 0.0001; *snf8* MO vs*. snf8* MO + *SNF8*^WT^, ^∗∗^p = 0.0024. In (D), n = 15 (not inj.), 12 (*snf8* MO), and 12 (*snf8* MO + *SNF8*^WT^). Two-way ANOVA with Tukey’s *post hoc* test (not inj. vs*. snf8* MO, ^∗∗∗∗^p < 0.0001; *snf8* MO vs*. snf8* MO + *SNF8*^WT^, ^∗∗∗∗^p < 0.0001). (E) Quantification of the dimension of the angle formed by the optic chiasm (OC) in the midline. n = 15 (not inj.), 9 (*snf8* MO), and 12 (*snf8* MO + *SNF8*^WT^). One-way ANOVA with Tukey’s *post hoc* test is used to assess statistical significance: not inj. vs*. snf8* MO, ^∗∗^p = 0.0039; *snf8* MO vs*. snf8* MO + *SNF8*^WT^, ^∗^p = 0.0463. In (C)–(E) data are expressed as boxplots with mean ± SEM.

We last examined the morphology of the extra-retinal optic nerve (ON) within the ventral brain. *snf8* morphant fish showed a reduced penetrance of morphological defects, resembling the condition observed in a large proportion of affected individuals (Figures 7A and 7B). We defined the conditions as “mild” (i.e., thin or slightly abnormal ON), “moderate” (thin or slightly abnormal ON with noticeable reduction of the retinal ganglion cell arborization field), and “severe” (severely reduced axonal scaffold, and extremely thin and short ON and/or exhibiting misrouted axons at the level of the OC). By quantifying both the extension of the ON and the thickness, we observed a statistically significant reduction in size, which was rescued in fish co-injected with *snf8* MO and WT *SNF8* (Figures 7A–7D).

In the ventral midline within the brain, as a result of a finely tuned growth and guidance mechanisms of crossing axons of the retinal ganglion cells, the optic chiasm (OC) shows a characteristic angle.^43^ A second defect involving the OC morphogenesis was observed in *snf8* morphant fish, especially in moderately or severely affected animals, which lost the typical angle of occurring in wild-type embryos. Again, injection of *SNF8* in *snf8* morphant fish was able to also rescue the OC phenotype (Figure 7E).

Taken together, the results proved pathogenicity of both the two tested variants (p.Tyr167Ter, family A; p.Val102Ile, families D–F) and definitively linked a defective Snf8 function to altered brain development and ON morphology.

## Discussion

As more and more gene-disease associations are made, the question of how to best define monogenic disorders with regard to phenotypic and genotypic variability has become of high importance. This discussion is often referred to as “lumper vs*.* splitter” dichotomy.^44^ In this case, lumpers prioritize similarities and thus allow more geno- and phenotypic variability in a monogenic disorder. In contrast, splitters define distinct clinical phenotypes as separate allelic disorders to emphasize defining characteristics. We provide evidence for an association of bi-allelic *SNF8* variants with a neurodevelopmental/neurodegenerative disease entity with a variable phenotype due to defective ESCRT-II function, at least in part ascribed to an altered autophagic flux. The broad phenotypic spectrum observed in the nine reported individuals ranged from a severe developmental and epileptic encephalopathy with leukoencephalopathy and early death to a milder phenotype comprising DD/ID and ON atrophy.

Specifically, the severely affected individuals presented with profound developmental and epileptic encephalopathy, while the mildly affected individuals showed involvement of the ON, although the young age of the individuals does not exclude a possible involvement of other organs at a later age. Severely affected individuals presented within the first weeks of life with muscular hypotonia and feeding difficulties and dysphagia requiring PEG feeding later. Brain imaging shows severe brain atrophy and leukoencephalopathy as well as corpus callosum aplasia or hypoplasia. EEGs were normal in early life, but individuals developed seizures at the age of ∼7–9 months with mostly myoclonic and focal tonic seizures and infantile spasms. EEG shows multifocal and generalized epileptic discharges further progressing to hypsarrythmia. From the severely affected cases, individuals A2 and C2 did not have seizures but seizure onset of individuals A1 and B1 indicates that they died too young to develop epilepsy. Both individuals A1 and B1 developed hyperreflexia and spasticity later in the course of the disease caused by degeneration of upper motor neurons.

The interfamilial variability and the consistent phenotype observed within families support the occurrence of a specific genotype-phenotype correlation in which all individuals harboring the missense variant p.Val102Ile in *trans* with a second variant (either truncating or missense, see Figure 1) show a milder phenotype. Clinical data as well as proteomics data revealed no significant reduction in ESCRT-II subunits protein levels in the mildly affected individuals D1 and E1, suggesting a hypomorphic effect of p.Val102Ile as compared to the variants identified in severely affected individuals. This hypothesis is also strengthened by the observation of two presumably healthy individuals with the respective variant in homozygous state—one affected by a different monogenic disorder and the other present in the gnomAD dataset. The short survival of the individual from the in-house database of the Institute of Human Genetics in Munich hampers the long-term assessment regarding a possible manifestation of ID and optic atrophy due to the homozygous c.304G>A (p.Val102Ile) variant later in life but proteomics indicate that the associated biological mechanism is not reduced ESCRT-II protein levels but likely reduced activity. Likely, the hypomorphic variant p.Val102Ile acts via a distinct mechanism independent of protein stability and formation of the ESCRT-II complex.

In the zebrafish model, knockdown of *snf8* resulted in a clear developmental phenotype, with involvement of brain development. Conversely to the wild-type mRNA of human *SNF8*, all disease-associated mutated alleles (*SNF8*^Y167∗;G191D^, *SNF8*^P79L;V102I^, *SNF8*^Y167∗^, and *SNF8*^V102I^) failed to rescue the global embryo phenotype, corroborating their pathogenicity. A more severe impact of the variants combinations characterizing subjects A1 and A2 (*SNF8*^Y167∗;G191D^, associated with early childhood death) compared to those found in subject F (*SNF8*^P79L;V102I^) was recapitulated and validated in fish by examining the effect of the variants on early lethality and embryo phenotype and is in line with a hypomorphic effect of the variant p.Val102Ile. The *in vivo* validation analysis supports the contribution of SNF8 function in embryo development and brain formation and corroborates both the pathogenicity and the variable impact on embryo development of a subset of variant combinations found in affected individuals, ultimately explaining the heterogeneous clinical spectrum of the disease. In-depth characterization of the transient zebrafish *snf8* loss-of-function model showed reduced brain size accompanied by axonal scaffold hypoplasia (including ON) as major features, which recapitulate traits observed in affected individuals. Of note, the role of the ESCRT-II complex in axonal growth in the context of retinal ganglion cell, which is emerging from our study, is supported by experiments in another *in vivo* vertebrate model, *Xenopus*. Indeed, LoF of the ESCRT-II Vps25 subunit in frogs leads to axonal growth defects and reduced ON diameter,^45^ resembling the phenotype observed in a subset of the affected individuals and in our zebrafish models of SNF8 LoF.

The finding of optic atrophy in the mildly affected individuals in the context of abnormal development of the optic system, suggesting optic hypoplasia, is puzzling as no visual abnormalities were found in the severely affected individuals. It is possible that defective SNF8 function results in a neuronopathy with high susceptibility of the optic nerve to degeneration whereas we assessed only neurodevelopment but not neurodegeneration upon MO-mediated knockdown of *snf8* in zebrafish with high susceptibility of the optic system to display abnormalities. On the other hand, corpus callosum (CC) agenesis or hypoplasia indicates a neurodevelopmental disease affecting CC formation as early as in the 10–18 weeks of gestation by impaired axonal pathfinding in the developing brain.^46^

Classification of white matter abnormalities are challenging in *SNF8*-associated developmental and epileptic encephalopathy. Clinical, *in vitro*, and *in vivo* data suggest a primary affection of neuronal cells rather than a pure myelin disorder. Hence, we believe that the leukoencephalopathy is best classified as a leuko-axonopathy due to early-onset neuronal degeneration.^47^

Individual A2 had pachygyria which typically occurs in neuronal migration disorders whereas all other individuals did not have MRI findings associated with impaired neuronal migration. Identification of more individuals with *SNF8*-associated disease and further studies in gyrencephalic animals will help to clarify whether pachygyria is indeed associated with a loss of *SNF8* or rather an unrelated feature.

Additionally, we cannot exclude that further delineation of the neurodevelopmental and neurodegenerative phenotype in the zebrafish model in a longitudinal analysis of stable LoF models and in mutants recapitulating the genotype of affected individuals might reveal substantial differences in residual SNF8 function on brain development and shed light on the hypothesized susceptibility of the ON to degeneration *in vivo*. Comparative assessment in other animal models would also contribute to better understanding the neurodevelopmental and neurodegenerative aspects associated with a loss of *SNF8*.

Our data derived from fibroblasts and human brain tissue samples show that the reported bi-allelic *SNF8* variants in family A lead to reduced protein levels of ESCRT-II subunits. Furthermore, patient-derived fibroblasts of individual A2 revealed that depletion of SNF8 results in accumulation of autolysosomes. Congruently, neuropathological evaluation also suggested impaired autophagy as observed by the accumulation of LC3-positive structures in neurons and glia cells. The most plausible explanation as to why ESCRT-II dysfunction would lead to accumulation of autolysosomes is that sorting of certain lysosomal hydrolases to the (auto)lysosome is affected. Consistent with this hypothesis, lysosomes with aberrant morphology were also detected in the affected-individual-derived fibroblasts. This is similar to defective sorting of hydrolases to the vacuole in yeast *vps22* mutants^5^ and would lead to impaired hydrolytic functions and thereby lower turnover of autolysosomes. Impaired autophagy due to defective ESCRT machinery subsequently resulting in a neurologic phenotype have previously been demonstrated in a conditional knockout mouse model of the ESCRT-associated protein hepatocyte growth factor-regulated tyrosine kinase substrate (HGS). In this model, *Hgs* knockout in the murine forebrain impaired autophagic flux, which led to the accumulation of ubiquitinylated proteins and ultimately resulted in the depletion of hippocampal neurons.^48^ Furthermore, defects in autophagic degradation were hinted as a possible pathomechanism in ALS due to variants in *CHMP2B* encoding an ESCRT-III subunit as the accumulation of p62-positive structures was observed in a fibroblast model as well as brain tissue samples of affected individuals.^12^^,^^19^

As quiescent neurons are especially sensitive to the accumulation of aberrant protein turnover, defects in autophagy have been recognized as a common patho-mechanism in multiple neurodegenerative diseases (including ALS, FTD, Huntington disease, and Parkinson disease as well as pediatric-onset neurodegenerative diseases).^49^^,^^50^^,^^51^^,^^52^^,^^53^ While the importance of autophagic turnover for neuron survival has been a subject of common interest, little is known about the role of autophagy in other cell populations of the central nervous system, specifically in oligodendrocytes forming the myelin of the CNS. Inhibition to the autophagic processes has recently been linked to an impaired myelination, for example knockdown of the autophagic regulator *Atg5* in oligodendrocytes results in thinner myelin sheaths compared to wild-type oligodendrocytes.^54^ One possible explanation is the resulting dysfunction in myelin compaction and the removal of excess cytoplasm through autophagy. Additionally, autophagy is hypothesized to influence oligodendrocyte differentiation.^54^ Thus, impaired autophagy might lead to additional dysfunctions in myelin production and/or compaction, which could explain the high degree of leukoencephalopathy and reduction in total cerebral white matter in the reported individuals.

As endosomal trafficking and autophagy are essential in most organ systems, it is to some extent surprising that defects in the ESCRT system are so far mostly linked to diseases manifesting in the central nervous system. The selective manifestation of ESCRT defects in the nervous system could be explained by cargo specificity or additional functions of ESCRT proteins in the brain. For example, ESCRT proteins have been shown to influence neuronal development including dendritogenesis,^55^ neuronal pruning,^56^^,^^57^ and axonal guidance.^45^ Our experimental data show that even in fibroblasts from severely affected individuals with drastic reduction of ESCRT-II subunits, there is some residual function of the ESCRT-II complex as EGFR degradation via MVBs was normal. Based also on the data derived from mRNA and protein analysis, one can hypothesize that the phenotypic differences observed in the individuals are a result of a more robust residual function of ESCRT-II in individuals harboring the hypomorphic p.Val102Ile variant.

In conclusion, we causally link the defective SNF8 function to disruption of the ESCRT-II complex, impaired autophagy, and a clinically variable neurodevelopmental/neurodegenerative disorder. The identification of additional affected individuals and further experimental studies directed to functionally characterize individual pathogenic variants will be crucial to clarify the clinical variability of this disorder and more accurately define the occurring genotype-phenotype correlations.

## Data and materials availability

All data needed to evaluate the conclusions in the paper are present in the paper and/or the supplemental methods. DNA and RNA sequencing as well as proteomics data are available upon request if in line with the written informed consents provided by the affected individuals or their legal guardians.

Variants were submitted to Clinvar with the following accession numbers: c.501C>A (GenBank: NM_007241.4) (p.Tyr167Ter), ClinVar: SCV004174808; c.572G>A (GenBank: NM_007241.4) (p.Gly191Asp), ClinVar: SCV004174809; c.236C>T (GenBank: NM_007241.4) (p.Pro79Leu), ClinVar: SCV004174810; c.623G>T (GenBank: NM_007241.4) (p.Arg208Leu), ClinVar: SCV004174811; c.423−1G>C (GenBank: NM_007241.4), ClinVar: SCV004174812; c.673_683delinsTGGA (GenBank: NM_007241.4) (p.Asp225TrpfsTer99), ClinVar: SCV004174813; c.304G>A (GenBank: NM_007241.4) (p.Val102Ile), ClinVar: SCV004174814.

## Web resources

ClinVar, https://www.ncbi.nlm.nih.gov/clinvar/
GenBank, https://www.ncbi.nlm.nih.gov/genbank/
OMIM, https://www.omim.org/

## Supplemental information

Document S1. Supplemental note, Figures S1–S18, Tables S1 and S2, and supplemental methods

Table S3. SNF8_clinical_details_20240119

Document S2. Article plus supplemental information
